# Epigenetic processes involved in response to pesticide exposure in human populations: a systematic review and meta-analysis

**DOI:** 10.1093/eep/dvae005

**Published:** 2024-04-20

**Authors:** Paula Rohr, Shimoyama Karen, Luiza Flávia Veiga Francisco, Marco Antônio Oliveira, Martins Fidelis dos Santos Neto, Henrique C S Silveira

**Affiliations:** Molecular Oncology Research Center, Barretos Cancer Hospital, Rua Antenor Duarte Vilela, 1331, B. Dr. Paulo Prata, Barretos, SP 14784-390, Brazil; Molecular Oncology Research Center, Barretos Cancer Hospital, Rua Antenor Duarte Vilela, 1331, B. Dr. Paulo Prata, Barretos, SP 14784-390, Brazil; Molecular Oncology Research Center, Barretos Cancer Hospital, Rua Antenor Duarte Vilela, 1331, B. Dr. Paulo Prata, Barretos, SP 14784-390, Brazil; Molecular Oncology Research Center, Barretos Cancer Hospital, Rua Antenor Duarte Vilela, 1331, B. Dr. Paulo Prata, Barretos, SP 14784-390, Brazil; Molecular Oncology Research Center, Barretos Cancer Hospital, Rua Antenor Duarte Vilela, 1331, B. Dr. Paulo Prata, Barretos, SP 14784-390, Brazil; Molecular Oncology Research Center, Barretos Cancer Hospital, Rua Antenor Duarte Vilela, 1331, B. Dr. Paulo Prata, Barretos, SP 14784-390, Brazil; Campus São Paulo, University of Anhanguera, São Paulo, SP 04119-901, Brazil

**Keywords:** pesticide exposure, epigenetic, DNA methylation, microRNA expression

## Abstract

In recent decades, the use of pesticides in agriculture has increased dramatically. This has resulted in these substances being widely dispersed in the environment, contaminating both exposed workers and communities living near agricultural areas and via contaminated foodstuffs. In addition to acute poisoning, chronic exposure to pesticides can lead to molecular changes that are becoming better understood. Therefore, the aim of this study was to assess, through a systematic review of the literature, what epigenetic alterations are associated with pesticide exposure. We performed a systematic review and meta-analysis including case-control, cohort and cross-sectional observational epidemiological studies to verify the epigenetic changes, such as DNA methylation, histone modification and differential microRNA expression, in humans who had been exposed to any type of pesticide. Articles published between the years 2005 and 2020 were collected. Two different reviewers performed a blind selection of the studies using the Rayyan QCRI software. Post-completion, the data of selected articles were extracted and analyzed. Most of the 28 articles included evaluated global DNA methylation levels, and the most commonly reported epigenetic modification in response to pesticide exposure was global DNA hypomethylation. Meta-analysis revealed a significant negative correlation between Alu methylation levels and β-hexachlorocyclohexane, *p*,*p'*-dichlorodiphenyldichloroethane and *p,p′*-dichlorodiphenylethylene levels. In addition, some specific genes were reported to be hypermethylated in promoter regions, such as *CDKN2AIGF2, WRAP53α* and *CDH1*, while *CDKN2B* and *H19* were hypomethylated due to pesticide exposure. The expression of microRNAs was also altered in response to pesticides, as miR-223, miR-518d-3p, miR-597, miR-517b and miR-133b that are associated with many human diseases. Therefore, this study provides evidence that pesticide exposure could lead to epigenetic modifications, possibly altering global and gene-specific methylation levels, epigenome-wide methylation and microRNA differential expression.

## Introduction

According to the US Environmental Protection Agency, pesticides are a large group of chemicals used to prevent, repel, destroy or mitigate unwanted living organisms There are many different types of pesticides developed to control several types of pests, such as herbicides, insecticides and fungicides, listed in order of greater use in Brazil, followed by many others that are used to a lesser degree [1]. These are subdivided according to their chemical groups, such as organochlorines, organophosphorus, carbamates, pyrethrin and pyrethroids [2]. Pesticides are widely used in agriculture (e.g. crops, cattle and forestry) and even in urban areas (e.g. gardening, vector control and industrial) [3]. Consequently, in addition to occupational exposure, humans may be exposed to such substances through numerous other forms, including through ingestion of contaminated food or water. Furthermore, the consequences of pesticide exposure on human health can vary from acute symptoms or even death, due to high-level exposure of pesticides; delayed health effects, such as reproductive, metabolic and respiratory disorders; neurotoxicity; carcinogenicity; and fetal developmental defects, over the lifespan of an individual [4]. Epidemiologic and experimental studies have identified several molecular mechanisms by which pesticides can induce toxicity in humans, including oxidative stress, endocrine disruption, genetic damage and epigenetic alterations [5].

Epigenetic modifications are mechanisms that regulate gene expression in response to endogenous and exogenous stimuli. These changes are heritable and can alter gene expression at the pre-transcriptional level, such as histone modification and DNA methylation, and at the post-transcriptional level, such as microRNA (miRNA) expression. DNA methylation is a non-genotoxic DNA modification consisting of the addition of a methyl group to the cytosines preceding guanines. When a modification occurs in a gene’s promoter region, it impedes the binding of activating transcription factors and triggers the formation of a closed chromatin structure through specific histone modifications. This prevents the RNA polymerase from binding to the DNA molecule and initiating the transcription of genes into messenger RNA (mRNA), which is a pivotal mechanism in gene silencing. On the other hand, a de-methylation can lead to gene activation. Generally, these changes occur in specific regions of the DNA, in CpG islands, but it is not restricted to these regions. Histones are proteins associated with DNA to form chromatin. Thus, covalent modifications (e.g. methylation and acetylation) to histones can lead to chromatin compaction changes resulting in either gene expression repression or activation. MicroRNAs are small non-coding RNAs that act by complementarily binding to the messenger RNA, inhibiting ribosomes from binding to it or even degrading the mRNA and preventing it from being translated [6].

All these mechanisms can alter the expression of many disease-related genes, the DNA repair and cell cycle-related genes, as well as in healthy and pathological aging [7]. Moreover, pesticide exposure has already been associated with many health issues; however, the molecular mechanisms by which this occurs are still unclear. Therefore, the aim of our systematic review was to provide a comprehensive overview of possible epigenetic effects induced by pesticide exposure in human populations.

## Materials and methods

A systematic literature search was conducted to collect studies that evaluated epigenetic changes, such as DNA methylation, histone modification and differential microRNA expression, in humans who had been exposed to any type of pesticide. This systematic review was performed according to the checklists and guidelines of the Preferred Reporting Items for Systematic Reviews and Meta-Analyses Statement 2020 [8].

### Data sources and searches

Article review involved extensive searches across major human health databases, including PubMed, Embase, and Cochrane and Biblioteca Virtual em Saúde (BVS—the Latin American Health Database). Searches covered articles published through December 2020. Considering the diverse terminologies used to describe the same epigenetic mechanism in this field, the search strategy aimed to encompass all relevant articles by combining a variety of search terms in titles and abstracts. This approach was implemented across PubMed, Embase and Cochrane databases to ensure comprehensive coverage. The search terms were entered exactly as follows: (Pesticides OR “Pesticide Exposure” OR “Exposure to Pesticides” OR Pesticide OR Agrochemical OR Herbicide OR Fungicide OR Insecticide OR Acaricide OR “Pesticide Applicators” OR Agricultural OR “Farmer workers”) AND (Epigenetics OR “Epigenetics Modifications” OR “Epigenetic Biomarkers” OR Methylation OR “DNA methylation” OR microRNA OR miRNA OR Histone OR “Histone Modifications” OR “Histone H3” OR “Histone H4” OR H3 OR H4 OR Epigenome OR “Epigenome wide studies association” OR Epigenetic OR “MicroRNA regulation” OR “MicroRNA expression” OR “DNA methylation profiles” OR “Gene-specific methylation” OR Epimutation OR “Epigenetic effects” OR “Circulating microRNAs” OR “MicroRNA profile” OR “Urinary microRNAs” OR “Potential biomarkers” OR “DNA methylation alterations” OR “MicroRNA profiling” OR “Epigenetic alterations” OR Acetylation OR “Global methylation” OR “DNA methylation alteration”). Despite using the same terms, the search strategy for the BVS database has a slightly different structure, as outlined in Supplementary Material 1.

### Eligibility criteria

In this systematic review were included observational studies, such as cross-sectional, and cohort studies, conducted in healthy individuals of any ethnicity, age or sex, published in English between January 2005 and December 2020. Accordingly, studies conducted with participants with known inherent/congenital or acquired genetic disorders were not eligible. Duplicate articles or incomplete texts were also excluded from our analysis.

### Selection process

All the articles were reviewed independently by two reviewers at all stages using the software Rayyan—Intelligent Systematic Review. An initial screening was performed in which titles and abstracts were read by all three reviewers to decide whether the articles met the inclusion or exclusion criteria of the systematic review. The same process was repeated using the full text of the remaining articles for the second screening.

### Data extraction

Datasheets were created containing information such as authors, type of study, journal and year of publication; characteristics of the study and population (sample size, ethnicity, mean age and the total number of exposed and unexposed); exposure (type of exposure, mean years of exposure, which pesticides and how exposure was assessed); and outcomes (epigenetic changes analyzed, method used to assess epigenetic changes and classification of results). Each eligible study was analyzed independently by two reviewers to complete the datasheets.

### Quality assessment

The methodological quality of the included articles was assessed using the National Heart, Lung, and Blood Institute (NHLBI) quality assessment tool (i.e. risk of bias). An NHLBI tool was used for observational cohort and cross-sectional studies. Two authors independently evaluated the selected articles as “yes,” “no,” “cannot determine,” “not reported” or “not applicable.”

These were discussed and used to formulate an overall rating for the quality of each study as “good,” “fair” or “poor.” Ratings were based on the number of quality assessment questions answered with “yes”: poor ≤6, fair >6 and <10 and good ≥10 questions.

### Data synthesis

A narrative (descriptive) synthesis of the results was performed. Due to the heterogeneity of the studies, different types of exposures, measurements and analyses, not all articles met the criteria for meta-analysis. Only studies that demonstrated a correlation coefficient between a pesticide or metabolite and Alu or long interspersed nuclear elements-1 (LINE-1) methylation levels were included in meta-analysis. Meta-analysis was performed when at least three articles evaluated the same pesticide or metabolite and the same effect. Among the included articles assessing global DNA methylation in the repetitive elements Alu and LINE-1, three articles had the same exposure, measurements and analysis. Thus, 6 different meta-analyses were performed for the correlation between each pesticide or metabolite concentration and repetitive sequence methylation. In the meta-analysis, random effects models and weighted effect sizes were used. The heterogeneity between studies was estimated using the Cochran *Q*-test and quantified by the inconsistence index (*I*-squared value). These analyses were performed using Comprehensive Meta-Analysis version 2.2.064.

## Results

### Study selection

**Figure 1:**
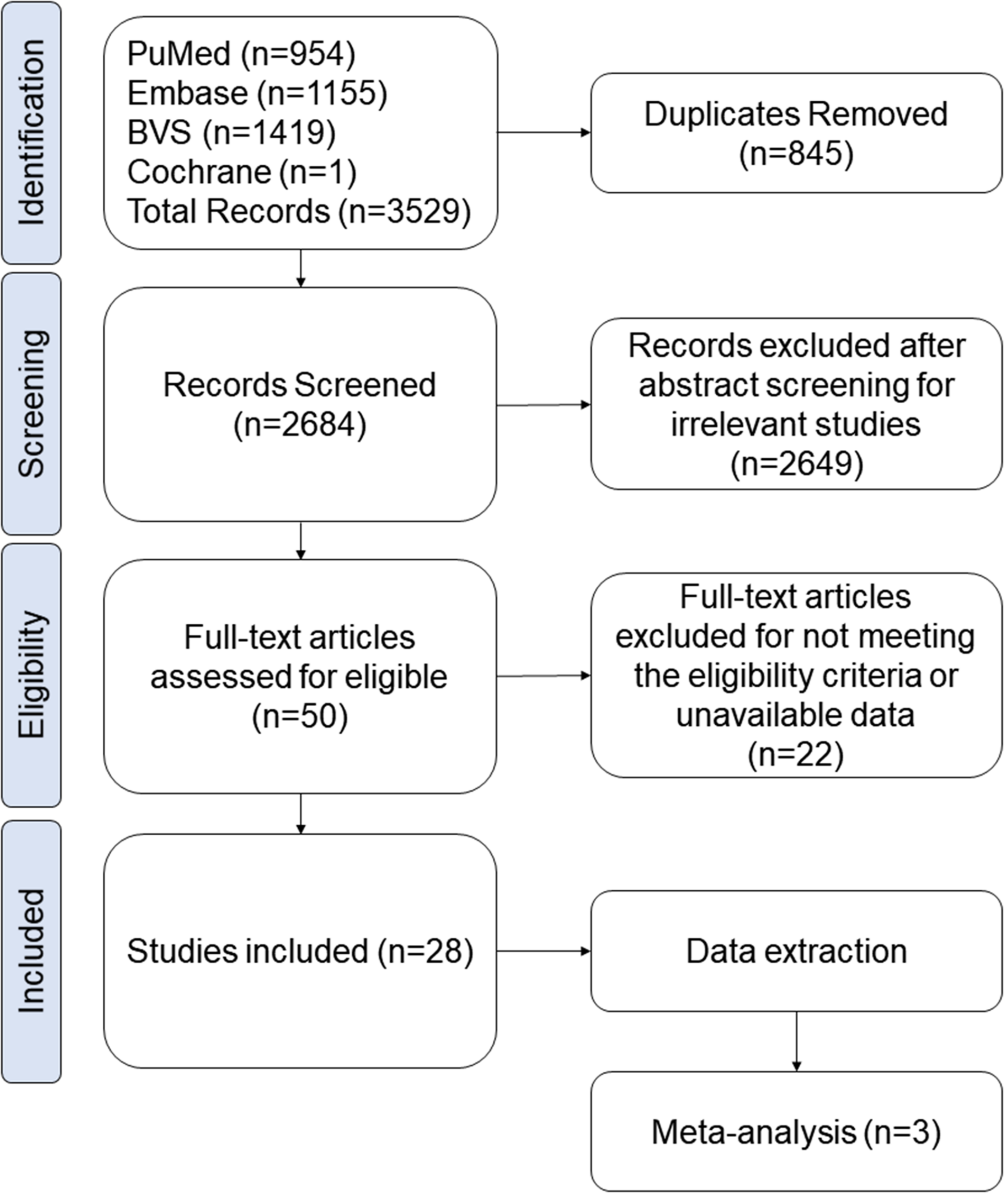
Flow diagram of manuscript selection and exclusion criteria in this review

The search resulted in 3529 articles found, 954 from PubMed, 1155 from EMBASE, 1419 from BVS and 1 from Cochrane, as shown in Fig. 1. Of these, 845 study duplicates were identified and removed. After screening titles and abstracts against the eligibility criteria, 2649 articles were excluded. Subsequently, 50 articles underwent full-text screening, of which 22 articles were deemed ineligible and removed. Finally, 28 articles fully met the eligibility criteria and were selected for data extraction (Fig. 1).

### Characteristics of the included studies

A total of 28 articles were included, and the majority (26 studies) were about DNA methylation, while only 2 were about microRNA differential expression and none were about histone modification. Among the DNA methylation studies, 14 were on global DNA methylation, 7 on DNA methylation arrays and 7 on gene-specific methylation. Global DNA methylation studies assessed the methylation levels at the repetitive sequences Alu and LINE-1 or at all 5-methyl-cytosine across the genome. The methodologies used to assess DNA methylation vary between pyrosequencing followed by polymerase chain reaction (PCR), liquid chromatography, high-performance liquid chromatography (HPLC) and luminometric methylation assay (LUMA). The assessment of DNA methylation arrays included studies that utilized the Illumina Infinium platform. The method used to assess microRNA expression in both studies was PCR array. In addition, most studies evaluated DNA methylation in peripheral blood.

### Overview of the epigenetic mechanisms related to pesticide exposure in human population

#### Global DNA methylation evaluated by repetitive sequences (Alu and LINE-1)

As described in Table 1, the most commonly reported epigenetic modification in response to pesticide exposure was global DNA hypomethylation [9–15]. On the other hand, only two articles reported DNA global hypermethylation [16, 17]. While Lind *et al*. (2013) [17] found that *p,p′*-dichlorodiphenylethylene (*pp'*-DDE) was associated with DNA hypermethylation, Benedetti *et al*. (2008) found DNA hypermethylation in individuals exposed to a complex mixture of pesticides [16].

**Table 1:** Articles assessing global DNA methylation in response to pesticide exposure

Reference	Study design	DNA methylation assessment	Methodology	Study population	Exposure assessment	Exposure	Outcome
Benitez-Trinidad *et al*., 2018 [9]	Cross-sectional	DNA methylation via LINE-1 in whole blood	Pyrosequencing	127 pesticide applicators and 63 healthy controls	Self-reported (questionnaire)	Mix of pesticides	DNA hypomethylation
Itoh *et al*., [10]	Cross-sectional	DNA methylation in peripheral leukocytes	LUMA (pyrosequencing)	403 Japanese women	High-resolution mass spectrometer with selected ion monitoring coupled to a gas chromatograph, based on isotope dilution mass spectrometry	β-HCH, *pp'*-DDE, *cis*-heptachlor epoxide, *trans*-nonachlor (*trans*-nonachlordane), *pp'*-DDD (dichlorodiphenyldichloroethane, *pp'*-DDT dichlorodiphenyltrichloroethane	Global hypomethylation
Kahl *et al*., 2018 [11]	Cross-sectional	Global DNA methylation in lymphocytes	Liquid chromatography by HPLC	56 tobacco farmers (21 males and 35 females) and 74 unexposed individuals (13 males and 61 females)	Self-reported (questionnaire)	HCH, HCB; heptachlor epoxide; oxy-chlordano; *trans*-nonachlor; *pp'*-DDE (dichlorodiphenyldichloroethylene) ; *pp'*-DDD; *pp'*-DDT; Mirex	Global Hypomethylation (*P* < 0.001)
Kahl *et al*., 2018 [12]	Cross-sectional	DNA Methylation in peripheral lymphocytes	Liquid chromatography by HPLC	40 non-exposed and 40 exposed (tobacco farmers) to pesticides	Self-reported (questionnaire)	OP	Global hypomethylation
Paredes-Céspedes *et al*., 2021 [13]	Cross-sectional	DNA methylation via LINE-1 in whole blood	Pyrosequencing	140 Huichol indigenous people from Nayarit, Mexico (117 FWs—59 women and 58 men—and 23 indigenous referents—19 women and 4 men) compared with a non-exposed Mestizo group of 47 individuals	OP metabolites measured in participants’ urine by gas chromatography coupled to mass spectrometry	*op'*-DDT, *pp'*-DDT, pp-DDE	Global hypomethylation (*P* < 0.01)
Rusiecki *et al*., 2008 [14]	Cross-sectional	DNA methylation via Alu and LINE-1 in whole blood	Pyrosequencing	71 Greenlandic Inuit (61 men and 9 women)	Pesticide measurement in participant’s plasma by gas chromatography	OP	Global hypomethylation
Rusiecki *et al*., 2017 [15]	Cohort	DNA methylation via LINE-1 in whole blood	Pyrosequencing	695 male AHS pesticide applicators (with high pesticide exposure)	Self-reported (questionnaire)	OP	Global hypomethylation
Benedetti *t al*., 2018 [16]	Cross-sectional	DNA methylation in whole blood	HPLC	137 individuals exposed to pesticides and 83 unexposed individuals	Self-reported (questionnaire)	Organochlorine (OC)	DNA hypermethylation
Lind *et al*., 2013 [17]	Cross-sectional	DNA methylation in whole blood	LUMA and pyrosequencing	524 individuals from the Vasculature in Uppsala (PIVUS - Prospective Investigation of Vasculature in Uppsala Seniors) study	Organochlorine serum measurement Micromass Autospec Ultima (Waters, Milford, MA, USA) HRGC/HRMS	OP temephos and chlorpyrifos	*p*,*p'*-DDE was associated with DNA hypermethylation
Alexander *et al*., 2017 [18]	Cross-sectional	DNA methylation via LINE-1 in leucocytes	Pyrosequencing	596 male AHS pesticide applicators	Self-reported (questionnaire)	Organochlorines hexachlorobenzene, *trans*-nonachlordane and *p*,*p'*-DDE	10 pesticides are associated with hypermethylation of LINE-1; 8 pesticides are associated with hypomethylation of LINE-1; an increase in 5 pesticides was associated with hypermethylation of LINE-1 (dose response); an increase in 3 pesticides was associated with hypomethylation of LINE-1
Huen *et al*., 2012 [19]	Cohort	DNA methylation via Alu and LINE-1 in umbilical cord blood representing fetal blood (at delivery) and whole blood (when they were 9 years old)	Pyrosequencing	358 pregnant women and their newborn children followed up after 9 years	Gas chromatography-mass spectrometry measurement of pesticides in the serum of participants (pregnant women)	Glyphosate (92.0%), flumetralin (28.6%), clomazone (20.6%), imidacloprid (19.0%) and sulfentrazone (17.4%)	Global DNA hypomethylation in fetal blood
Kim *et al*., 2010 [20]	Cross-sectional	DNA methylation via Alu and LINE-1 in whole blood	Pyrosequencing	86 healthy Koreans (34 males and 52 females)	Measurement of pesticides in participant’s serum	A complex mixture of pesticides (including glyphosate-based herbicides)	Global DNA hypomethylation (associations found only for POP concentrations and Alu methylation)
Kim *et al*., 2018 [21]	Cohort	DNA methylation by LINE-1 in placental tissue adjacent to the umbilical cord insertion	Pyrosequencing	148 healthy pregnant women	High-resolution gas chromatography measurement of serum OC pesticide levels coupled to a HRGC/HRMS	OPs (glyphosate-based herbicides), dithiocarbamates [Mancozeb (fungicide)], inorganic compounds (magnesium, aluminum, phosphide aluminum phosphide—insecticide) and cupric oxide (copper—fungicide)	β-HCH was associated with a decrease in LINE-1 methylation
Lee *et al*., 2017 [22]	Cross-sectional	DNA methylation via Alu and LINE-1 in peripheral leukocytes	Pyrosequencing	444 healthy Koreans (253 males and 191 females)	Measurement of pesticides in participant’s serum by gas isotope dilution method chromatography/high-resolution mass spectrometry	*op'*-DDT, *pp'*-DDT, *pp'*-DDE, *trans*-nonachlor, *cis*-nonachlor, oxy-chlordano, HCB, Mirex, HCH	Global DNA hypomethylation in the Alu assay for men and global DNA hypermethylation in the LINE-1 assay for women

Abbreviations: AHS, Agricultural Health Study; HRGC/HRMS, high-resolution gas chromatography coupled with high-resolution mass spectrometry; OC, organochlorine.

Some studies evaluated LINE-1 and Alu methylation as surrogate markers for global DNA methylation. The findings are variable depending on the specific pesticide exposure as well as each transposon analyzed, as shown in Table 1 [18–22].

#### Global DNA methylation meta-analysis

We performed six different meta-analysis of the eligible studies in this systematic review, examining three substances, pesticide or metabolite [β-hexachlorocyclohexane (β-HCH); *p,p′*-dichlorodiphenyldichloroethane (*pp'*-DDT) and *p*,*p′*-dichlorodiphenyldichloroethylene (*pp'*-DDE)] and their effects in the methylation levels in each of the repetitive sequences, Alu and LINE-1. In each meta-analysis, three studies that presented the correlation coefficient between the metabolite and in Alu or LINE-1 methylation levels were included.

**Figure 2:**
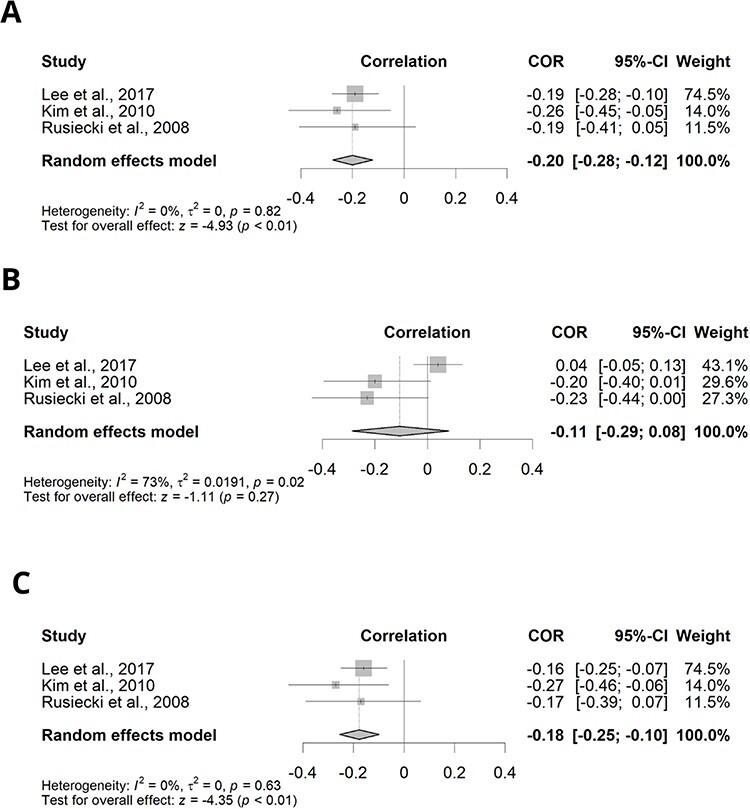
Correlation between specific pesticide or metabolite exposure and Alu methylation. Diamonds represent the estimated correlation; horizontal lines represent the 95% CIs. (A) β-HCH, (B) *pp'*-DDT and (C) *pp'*-DDE

The three meta-analyses that considered Alu methylation levels as an end point observed a negative correlation with β-HCH, *pp'*-DDT and *pp'*-DDE levels (Fig. 2). In the meta-analysis for β-HCH, the studies showed low heterogeneity (*I*^2^ = 0.000, *P* = 0.824) and a negative correlation between pesticide concentration and Alu methylation levels (−0.200; 95% confidence interval (CI): −0.276, −0.122; *P* < 0.001) (Fig. 2A). While the meta-analysis for *pp'*-DDT showed high heterogeneity (*I*^2^ = 73.197, *P* = 0.024), and although there was a negative correlation between pesticide concentration and Alu methylation levels, it was not statistically significant (−0.109; 95% CI: −0.300, 0.090; *P* = 0.282) (Fig. 2B).The meta-analysis for *pp'*-DDE showed low heterogeneity (*I*^2^ = 0,000, *P* = 0,626) and an inverse correlation between this metabolite concentration and Alu methylation levels (−0.177; 95% CI: −0.254, −0.098; *P* < 0.001) (Fig. 2C).

**Figure 3:**
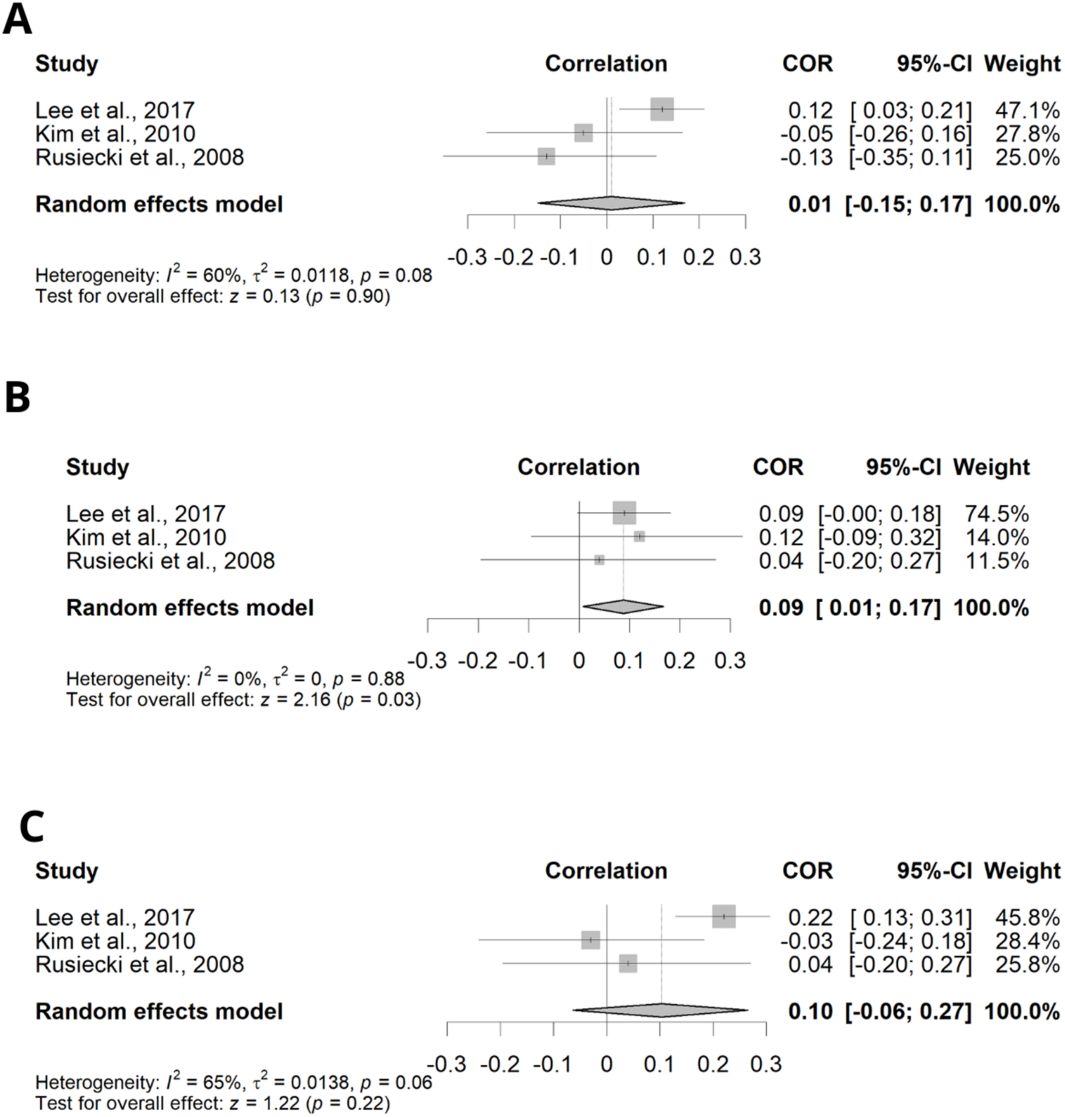
Correlation between specific pesticide exposure and LINE-1 methylation. Diamonds represent the estimated correlation; horizontal lines represent the 95% CIs. (A) β-HCH, (B) *pp'*-DDT and (C) *pp'*-DDE

On the other hand, the three meta-analyses that considered LINE-1 methylation levels as an end point showed a discrepant result (Fig. 3). While the meta-analysis on β-HCH and *pp'*-DDE showed no correlation between pesticide or metabolite concentration and LINE-1 methylation levels (Fig. 3A and C). The meta-analysis for β-HCH showed moderate heterogeneity among the studies (*I*^2^ = 60.487, *P* = 0.080) and correlation between pesticide concentration and LINE-1 methylation levels of 0.009 (95% CI: −0.153–0.170; *P* = 0.913) (Fig. 3A). For *pp'*-DDE, the studies showed moderate heterogeneity (*I*^2^ = 65.117, *P* = 0.057) and correlation between metabolite concentration and LINE-1 methylation levels of 0.102 (95% CI: −0.072–0.269; *P* = 0.249) (Fig. 3C). Additionally, the meta-analysis for *pp'*-DDT also showed low heterogeneity (*I*^2^ = 0.000, *P* = 0.884) and a correlation between pesticide concentration and LINE-1 methylation levels (0.088; 95% CI: 0.008–0.168; *P* = 0.031) (Fig. 3B).

#### Gene-specific methylation

Of the articles included in the review, seven reported modifications in gene-specific methylation profile caused by pesticide exposure (Table 2). These studies analyzed methylation of different genes associated with different pathways.

**Table 2:** Articles assessing gene-specific methylation in response to pesticide exposure

Reference	Study design	DNA methylation assessment	Genes	Gene region	Methodology	Study population	Exposure assessment	Exposure	Outcome
Rusiecki *et al*., 2017 [15]	Cohort	Gene-specific methylation in whole blood	MGMT, GSTp1	Promoter	Pyrosequencing	695 male AHS pesticide applicators	Self-reported (questionnaire)	OP pesticides (OPs)	MGMT hypomethylation, GSTp1 hypermethylation
Kim *et al*., 2018 [21]	Cohort	Gene-specific methylation in the placenta	H19, IGF12	3 CpG sites within IGF2 DMR0, 8 CpG sites within imprinting control region	Pyrosequencing	109 Korean mother–child pairs	19 serum measurement of OCs by (HRGC/HRMS)	*p*,*p′*-DDE, *p*,*p′*-DDT, β-HCH, *trans*-nonachlordane	IGF2 hypermethylation H19 hypomethylation
Herrera-Moreno *et al*., 2019 [23]	Cross-sectional	Gene-specific methylation in whole blood	CDKN2A (p16) and CDKN2B (p15)	CDKN2B (5 CpG sites in the 5′ UTR region) CDKN2A (6 CpG sites in promoter	Pyrosequencing	186 urban pesticide applicators and 102 controls	Measurement of urinary dialkylphosphates, OP metabolites (including dimethylphosphate, dimethylthiophosphate, dimethyl dithiophosphate, diethyl phosphate, diethylthiophosphate and diethyldithiophosphate) and a structured questionnaire using gas chromatography coupled to a mass spectrometer by selective ion monitoring	OP pesticides	CDKN2B hypomethylation, CDKN2A hypermethylation
Ratna *et al*., 2020 [24]	Cross-sectional	Gene-specific methylation in whole blood	p16 (CDKN2A)	Promoter	Methylation-specific PCR	78 farmers	Self-reported (questionnaire)	Pyrethroids, OPs, carbamates, macrocyclic lactones, neonicotinoids	21 individuals with p16 methylated
Park *et al*., 2015 [25]	Cross-sectional	Gene-specific methylation in peripheral leucocytes	MGMT	Promoter	Methylation-specific PCR	368 Koreans	Serum OC concentrations measured by GC-HRMS (gas chromatography-high-resolution mass spectrometry)	*pp'*-DDE, *pp'*-DDT, oxychlordane, *trans*-nonachlor, heptachlor epoxide, β-hexachlorocyclohexane, HCB, Mirex	MGMT hypermethylation
Lee *et al*., 2017 [26]	Cross-sectional	Gene-specific methylation in whole blood	CDH1		Methylation-specific PCR	364 healthy Koreans	High-resolution gas chromatographic measurement of serum OC pesticide levels mass spectrometry (GC/HRMS)	*pp'*-DDE, *pp'*-DDT, oxychlordane, *trans*-nonachlor, heptachlor epoxide, β-HCH, hexachlorobenzene, Mirex	CDH1 methylated
Paredes-Céspedes, *et al*., 2019 [27]	Cross-sectional	Gene-specific methylation in whole blood	WRAP53α	3 CpGs between exon 1α and intron 1α	Pyrosequencing	444 individuals (91 mestizo controls, 164 mestizo urban sprayers and 189 indigenous controls)	Dialkyl phosphate metabolite measurement (dimethylphosphate, dimethylthiophosphate, dimethyl dithiophosphate, diethyl phosphate, diethylthiophosphate and diethyldithiophosphate)	OP insecticides with temephos and chlorpyrifos as the main active ingredients and pyrethroids with deltamethrin as the active ingredient	WRAP53α hypermethylation

Similar results were found for *CDKN2A*, which showed hypermethylation in the promoter region due to pesticide mixture exposure in two studies [23, 24]. Herrera-Moreno *et al*. (2019) likewise reported CDKN2B hypomethylation in the 5ʹ untranslated region (UTR) region due to pesticide exposure [23].

Results regarding O^6^-methylguanine-DNA methyltransferase (*MGMT*) methylation are controversial, with two studies evaluating MGMT promoter region methylation levels in relation to pesticide exposure. While Park *et al*. (2015) demonstrated *MGMT* hypermethylation in individuals with detected organochlorides, Rusiecki *et al*. (2017) reported *MGMT* hypomethylation and *GSTP1* hypermethylation in individuals exposed to organophosphate (OP) pesticides [15, 25]. One study reported that DDT and OC exposures were associated with *IGF2* hypermethylation as well as *H19* hypomethylation [21]. Lee *et al*. (2017) reported *CDH1* promoter methylation in 78.3% of the 364 subjects exposed to OCs [26]. OCs were found to be significantly higher in subjects with CDH1 methylation. Exposure to OPs and pyrethroids results in *WRAP53α* hypermethylation [27].

#### DNA methylation arrays

The current study included seven DNA methylation arrays that analyzed the association between pesticide exposure and DNA methylation (Table 3). All DNA methylation arrays used the Infinium Human Methylation 450 BeadChip Array (Illumina). Most of the included studies were cross-sectional (*n* = 5), with two being cohort studies. Five of the selected studies assessed methylation in whole blood cells [28–32], one in cord blood cells [32], one in placental tissue [33] and one in sperm [34].

**Table 3:** Articles assessing DNA methylation arrays in response to pesticide exposure

Reference	Study design	DNA methylation assessment	Genes	Methodology	Study population	Exposure assessment	Exposure	Outcome
Furlong *et al*., 2020 [28]	Cross-sectional	DNA methylation arrays in whole blood		Illumina Infinium 450k platform	237 North Americans	California Pesticide Use Registry and geospatial-based system to assign exposure status to all controls	Pyrethroids	415 CpG sites were differentially methylated
Howard *et al*., 2016 [29]	Cross-sectional	DNA methylation arrays in whole blood		Illumina Infinium Human Methylation 450k BeadChip	83 FWs and 60 NFWs	No exposure assessment	Pesticides	Methylation at 36 CpG sites, located in or near 72 genes, hypermethylation in FWs and hypomethylation in NFWs
Lind *et al*., 2017 [30]	Cross-sectional	DNA methylation arrays in whole blood	CYP2B6 gene	Illumina Infinium Human Methylation 450k BeadChip	1016 elderly individuals from the Prospective Investigation of the Vasculature in Uppsala Senoirs (PIVUS) study	Serum measurement via HRGC/HRMS	*p*,*p′*-DDE	CYP2B6 methylation
van Der Plaat *et al*.,2017 [31]	Cross-sectional	DNA methylation arrays in blood		Illumina Infinium Human Methylation 450k arrays	1656 subjects with occupational exposure to pesticides	ALOHA+ Job Exposure Matrix (JEM)	Pesticides (sup. 1)	Differential DNA methylation of 31 CpGs annotated to 29 genes
Yu *et al*., 2018 [32]	Cohort	DNA methylation arrays in cord blood	BRCA1	Illumina Infinium Human Methylation 450K BeadChip	150 mother–newborn pairs	Umbilical Cord Detection by capillary gas chromatography	DDT and 6 homologs	1131 differentially methylated CpG sites, 690 hypermethylated, 441 hypomethylated, 598 unique genes, BRCA1 hypermethylation
Ouidir *et al*., 2020 [33]	Cohort	DNA methylation arrays in placenta	GMIP, C6orf217, RBM39, THNSL1, HIST1H2BI, SH3PXD2B, ZNF471, QRFP, FASN, PIGT, GGPS1.ARID4B, ALG10B, LMX1A, AKNA	tra	260 pregnant women	OC measurement in participant’s plasma	HCB, *trans*-nonachlor, *p*,*p′*-DDE	OCs associated with methylation at 14 CpG sites
Kelsey *et al*., 2019 [34]	Cross-sectional	DNA methylation arrays in sperm	TEAD3 gene had four distinct CpG sites that showed loss of DNA methylation in association with dioxin exposure. AND H19	Illumina Infinium Human Methylation 450k BeadChip	37 participants of the Air Force Health Study	Serum dioxin levels	Agent Orange	36 gene regions, including the region of the imprinted gene H19, found to have altered DNA methylation associated with high exposure compared to the low exposure group

Abbreviations: PIVUS, Prospective Investigation of the Vasculature in Uppsala Seniors; AFHS, Air Force Health Study; HRGC/HRMS, high-resolution gas chromatography coupled with high-resolution mass spectrometry.

Ouidir *et al*. (2020) found that OCs (hexachlorobenzeno (HCB), *trans*-nonachlor or *p*,*p′*-DDE) were associated with methylation at 14 CpG sites in the placenta of pregnant women who had been exposed, and the sum of OCs concentrations was associated with 25 CpG sites [33]. Yu *et al*. (2018) identified 1131 significantly different methylated CpG sites, with 690 hypermethylation sites and 441 hypomethylation sites in cord blood DNA methylation levels between DDE-exposed and non-exposed groups [32]. Lind *et al*. (2017), in turn, conducted a population-based Prospective Investigation of the Vasculature in Uppsala Seniors to investigate methylation levels after exposure to *p*,*p'*-DDE. The results showed a significant relationship between *p*,*p′*-DDE serum levels and cg27089200 methylation (located 7 kb downstream of the *CYP2B6* gene) in blood samples [30].

Furlong *et al*. (2020) analyzed individuals with chronic environmental exposure to pyrethroid and found that 415 CpG sites were differentially methylated [28]. In another investigation, Howard *et al*. (2016) compared the change in DNA methylation from whole blood between groups of farmworkers (FWs) and non-farmworkers (NFWs) and identified differences in 36 CpG sites between the two groups [29].

Van Der Plaat *et al*. (2017) observed differential DNA methylation of 31 CpGs in individuals with high pesticide exposure [31]. Assessing sperm samples from military veterans of Operation Ranch Hand exposed to Agent Orange, Kelsey *et al*. (2019) associated loss of DNA methylation in four different CpG sites of the *TEAD3* gene with dioxin exposure. In addition, by assessing regional DNA methylation changes, 36 gene regions, including the region of the imprinted gene H19, were found to have altered DNA methylation associated with high dioxin exposure compared to the low dioxin exposure group [34].

#### MicroRNA differential expression

The two studies evaluating microRNA expression are shown in Table 4. In a cross-sectional study of 34 individuals intentionally poisoned with Methamidophos, 37 miRNAs were found to be significantly different in the serum samples of poisoned patients in comparison to healthy controls, of which 29 microRNAs were found to be upregulated and 8 were found to be downregulated. In addition, further functional analysis indicated that many pathways potentially regulated by these miRNAs are involved in skeletal muscle, nervous system and heart disorders [35]. A cohort of mother–child FW pairs exposed to OP pesticides and NFW pairs was studied by measuring urinary microRNA profiles [36]. Significant differences in miRNA profiles were found between adult FWs and NFWs and also between seasons. During the post-harvest season, six miRNAs were identified as being positively associated with FWs (miR-223, miR-518d-3p, miR-597, miR-517b, miR-133b and miR-28-5p upregulated). For five of these miRNAs (miR-223, miR-518d-3p, miR-597, miR-517b and miR-133b), the expressed levels exhibited a trend toward a positive dose–response relationship with metabolites of OP pesticides among FWs [36].

**Table 4:** Articles assessing MicroRNA differential expression in response to pesticide exposure

Reference	Study design	microRNA expression assessment	Methodology	Study population	Exposure assessment	Exposure	Results
Yuan *et al*., 2018 [35]	Cross-sectional	Assessment of serum microRNA expression	TaqMan Human MicroRNA Array analysis	34 individuals with intentional acute oral OP poisoning	Self-reported	Organophosphorus pesticide Methamidophos	37 miRNAs were significantly different in the sera of poisoned patients compared to the healthy controls, including 29 miRNAs that were upregulated and 8 miRNAs that were downregulated
Weldon *et al*., 2016 [36]	Cohort	Assessment of microRNA expression in urine	TaqMan Array Human miRNA Panel A (4398977) covering 384 human miRNAs	Mother–child pair FWs (*n* = 16) and half non- FWs (*n* = 11).)	OP metabolites measured in urine samples via HPLC-MS/MS	OP pesticides	During the post-harvest season, six miRNAs were found to be positively associated with FW status. The expression of five of these miRNAs revealed a trend toward a positive dose–response relationship with OP pesticide metabolites in farm workers

Abbreviations: HPLC-MS/MS, HPLC coupled to mass detection; miRNA, microRNAs.

## Discussion

Exposure to pesticides has multiple effects on human health. Recent studies have identified epigenetic alterations as one of these effects. In the present systematic review study, the epigenetic processes involved in response to pesticide exposure in the human population were analyzed.

In this review, we systematically summarized the existing evidence on the association between human pesticide exposure and epigenetic alterations. Following data collection, a meta-analysis was performed to estimate common associations between specific pesticide exposures and global DNA methylation.

Even though the exposure to many different pesticides was assessed in the studies included in this systematic review, only 6 meta-analyses were performed considering β-HCH, *pp'*-DDT and *pp'*-DDE pesticides or metabolite levels and global methylation levels. The Alu methylation level showed a negative correlation with β-HCH, *pp'*-DDT and *pp'*-DDE metabolite levels in the meta-analysis, while no statistical significance was found for LINE-1 methylation and the same metabolites.

Although both Alu and LINE 1 methylation levels are markers of global methylation patterns, many studies have not shown concordant methylation levels between the two of them. As was shown by Lee *et al*. (2017) [22], in their analysis of both men and women exposed to persistent organic pollutants (POPs). The study found that global DNA hypomethylation in the Alu assay for men and global DNA hypermethylation in the LINE-1 assay for women was associated with POPs exposure, also demonstrating differences between the genders [22]. While Rusiecki *et al*. (2008) observed a significant difference between genders for Alu and LINE-1, they did not find an association between DNA methylation and POPs differed when we stratified linear regression analyses on sex [14]. On the other hand, Kim *et al*. (2010) did not observe differences between genders [20].

To identify CpG site modifications associated with phenotypes, DNA methylation arrays are a powerful methodology able to simultaneously evaluate a large number of CpGs. Our systematic review included seven DNA methylation arrays that examined the effects of exposure to different pesticides. Thus, the findings presented were varied. The diversity of the populations studied, particularly with regard to the substance to which they were exposed, may explain the different results found in each of the studies. In addition, alterations in CpG methylation levels could also be evaluated using gene-specific methylation studies. While this type of study may offer less comprehensive coverage of regions, it has the potential to yield results similar to those of DNA methylation arrays studies. For example, a study using DNA methylation arrays conducted in USA found *H19* hypomethylation levels in healthy individuals exposed to dioxin, consistent with OC pesticides levels observed in a gene-specific methylation study of healthy pregnant Koreans [21, 34].

MiRNAs play a role in the regulation of numerous cellular processes, which include apoptosis, cell differentiation, development, proliferation and tumor growth. MicroRNA expression may be regulated by xenobiotic exposure such as pesticides [36]. MicroRNA expression studies evaluating the effects of pesticide exposure effects are limited. This review included only two studies that analyzed different populations. One study evaluated microRNA expression profiling in human acute organophosphorus poisoning, and the other study observed miRNA profiles in archived urine samples of FWs. Both studies observed differential microRNA expression as a result of pesticide exposure or poising. The upregulation of the microRNAs, miR-223 and miR-28-5p, in the exposed groups was common to both studies [35, 36]. miR-223 has been described as a key regulator of innate immune response in respiratory diseases, a protective factor against atherosclerosis and a potential biomarker for breast cancer [37–39]. Moreover, miR-28-5p has been demonstrated as a potential biomarker for various diseases such as unstable angina, colon cancer, gastrointestinal cancer and prostate cancer [40–43]. Furthermore, this microRNA has been associated with environmental contaminants such as PM2.5 exposure and blood metal levels [44, 45].

Several limitations should be considered when interpreting our results. Despite following the guidelines of the Preferred Reporting Items for Systematic Reviews and Meta-Analyses Statement 2020, which included a well-defined search strategy, keywords and eligibility criteria, we observed a discrepancy between the number of articles identified in the searches and the number of articles that were ultimately included in our systematic review. It is evident that several articles were excluded due to the methodology used, which excluded articles with duplicates in the databases. Additionally, the peer review followed strict eligibility criteria, including only studies with healthy exposed individuals. This may explain the final number of articles selected in the systematic review. Furthermore, a substantial limitation of this review was the considerable heterogeneity in the types of pesticides to which the populations were exposed. The same applies to the heterogeneity of ethnicities and genders of exposed individuals, in addition to the different methods of exposure assessment, which ranged from self-report to quantification of specific pesticides detected in participants’ body fluids. Such heterogeneities were due to our study design, which included studies that evaluated individuals exposed to any pesticide. The diversity of epigenetic modifications analyzed in the studies included was another significant limitation of our review. This diversity, along with the different types of pesticides as well as the quantities to which individuals are exposed, made it difficult to observe more robust results.

Another limitation that can be considered in this study concerns the various methodologies used to analyze DNA methylation on a large scale. While our analysis did not exclude any particular DNA methylation methodologies based on eligibility criteria, the studies included in this systematic review primarily employed DNA methylation arrays, specifically utilizing the Illumina Infinium platform. It is worth noting that microarrays typically only analyze ∼2% of the genome. As a result, studies utilizing methodologies such as MeDip and BS-Seq, which, respectively, analyze 90% and 50% of CpG sites across the genome, were not selected for inclusion in our study, despite being considered genome-wide technologies. Additionally, microarrays tend to exhibit a bias toward detecting hypermethylation compared to hypomethylation, as they rely on comparisons of DNA methylation levels with a reference control group, potentially leading to inaccuracies in certain contexts.

Furthermore, the majority of studies included in this systematic review focused on analyzing epigenetic alterations in blood cells. One study examined DNA methylation levels in sperm, while others investigated methylation patterns in placental tissue. Consequently, some of these studies may involve a mixture of cell types, such as those observed in blood or placental tissue. This heterogeneity in cell composition can complicate the interpretation of epigenetic changes, as differences in the proportions of various cell types may confound the analysis. Therefore, the potential impact of this cell type heterogeneity should be recognized as an additional limitation of our study.

Moreover, it is essential to acknowledge that cell differentiation, which is driven by epigenetic modifications, can significantly influence DNA methylation patterns. Unfortunately, the studies in our analysis did not systematically account for differences in methylation levels between different cell types. Despite these limitations, it is important to emphasize that the conclusions drawn in the present manuscript remain unaffected. However, these limitations should be carefully considered when interpreting the results and assessing their broader implications.

## Conclusion

Despite the limitations stated, our review did provide evidence that pesticide exposure could lead to epigenetic modifications, possibly altering global and gene-specific methylation levels, epigenome-wide methylation and microRNA differential expression. The meta-analysis revealed a significant negative correlation between Alu methylation levels and β-HCH, *pp'*-DDT and *pp'*-DDE levels. In addition, some specific genes were reported to be hypermethylated in promoter regions, such as *CDKN2A, IGF2, WRAP53α* and *CDH1*, while *CDKN2B* and *H19* were hypomethylated due to pesticide exposure. The expression of microRNAs was also altered in response to pesticides, as miR-223, miR-518d-3p, miR-597, miR-517b and miR-133b that are associated with many human diseases. Nevertheless, these epigenetic modifications are results of specific pesticide exposure and may be linked to health effects.

## Supplementary Material

dvae005_Supp

## Abbreviations

β-HCH  β-Hexachlorocyclohexane

LUMA  Luminometric methylation assay

mRNA  Messenger RNA

miRNA  MicroRNA

*pp'*-DDE  *p,p′*-Dichlorodiphenyldichloroethylene

*pp'*-DDT  *p,p′*-Dichlorodiphenyldichloroethane
