# Supplementary material for: Epigenetic processes involved in response to pesticide exposure in human populations: a systematic review and meta-analysis
Source: Environ Epigenet. 2024 Apr 20;10(1):dvae005. doi: 10.1093/eep/dvae005 (PMC11110075; doi:10.1093/eep/dvae005)
Supplement: dvae005_Supp [file dvae005_supp.zip › Supplementary Material - BVS Strategy.docx]

[BVS Strategy- Supplementary data] (ti:Pesticides OR ti:"Pesticide Exposure" OR ti:“Exposure to Pesticides” OR ti:Pesticide OR ti:Agrochemical OR ti:Herbicide OR ti:Fungicide OR ti:Insecticide OR ti:Acaricide OR ti:"Pesticide Applicators" OR ti:Agricultural OR ti:"Farmer workers" OR ab:Pesticides OR ab:"Pesticide Exposure" OR ab:“Exposure to Pesticides” OR ab:Pesticide OR ab:Agrochemical OR ab:Herbicide OR ab:Fungicide OR ab:Insecticide OR ab:Acaricide OR ab:"Pesticide Applicators" OR ab:Agricultural OR ab:"Farmer workers") AND (ti:Epigenetics OR ti:"Epigenetics Modifications" OR ti:"Epigenetic Biomarkers" OR ti:Methylation OR ti:"DNA methylation" OR ti:microRNA OR ti:miRNA OR ti:Histone OR ti:"Histone Modifications" OR ti:"Histone H3" OR ti:"Histone H4" OR ti:H3 OR ti:H4 OR ti:Epigenome OR ti:"Epigenome wide studies association" OR ti:Epigenetic OR ti:"MicroRNA regulation" OR ti:"MicroRNA expression" OR ti:"DNA methylation profiles" OR ti:"Gene-specific methylation" OR ti:Epimutation OR ti:"Epigenetic effects" OR ti:"Circulating microRNAs" OR ti:"MicroRNA profile" OR ti:"Urinary microRNAs" OR ti:"Potential biomarkers" OR ti:"DNA methylation alterations" OR ti:"MicroRNA profiling" OR ti:"Epigenetic alterations" OR ti:Acetylation OR ti:"Global methylation" OR ti:"DNA methylation alteration" OR ab:Epigenetics OR ab:"Epigenetics Modifications" OR ab:"Epigenetic Biomarkers" OR ab:Methylation OR ab:"DNA methylation" OR ab:microRNA OR ab:miRNA OR ab:Histone OR ab:"Histone Modifications" OR ab:"Histone H3" OR ab:"Histone H4" OR ab:H3 OR ab:H4 OR ab:Epigenome OR ab:"Epigenome wide studies association" OR ab:Epigenetic OR ab:"MicroRNA regulation" OR ab:"MicroRNA expression" OR ab:"DNA methylation profiles" OR ab:"Gene-specific methylation" OR ab:Epimutation OR ab:"Epigenetic effects" OR ab:"Circulating microRNAs" OR ab:"MicroRNA profile" OR ab:"Urinary microRNAs" OR ab:"Potential biomarkers" OR ab:"DNA methylation alterations" OR ab:"MicroRNA profiling" OR ab:"Epigenetic alterations" OR ab:Acetylation OR ab:"Global methylation" OR ab:"DNA methylation alteration")
